# Impact of Scaling and Root Planing on Salivary and Serum Prolactin Levels in Patients With Periodontitis

**DOI:** 10.7759/cureus.93161

**Published:** 2025-09-24

**Authors:** Nileena Dilip, Nisha K J

**Affiliations:** 1 Periodontics, Vydehi Institute of Medical Sciences and Research Centre, Bangalore, IND

**Keywords:** biomarker, hormone, periodontitis, prolactin, saliva, serum

## Abstract

Introduction

Prolactin (PRL), though primarily a lactogenic hormone, additionally acts as a pro-inflammatory mediator produced by the pituitary and immune cells during inflammatory responses. Elevated PRL levels have been observed in autoimmune and inflammatory conditions, including periodontitis. This study aims to compare salivary and serum PRL levels in health and periodontitis and to evaluate the effect of non-surgical periodontal therapy on these levels.

Materials and methods

Sixty participants were divided into two groups: 30 healthy individuals (Group 1) and 30 patients with generalized Stage II to IV periodontitis (Group 2). Baseline saliva and serum samples were collected from all subjects, along with clinical periodontal assessments. All participants underwent scaling and root planing (SRP) and received oral hygiene instructions. Group 2 was re-evaluated three months post-treatment, with follow-up sample collection. PRL levels were measured using an enzyme-linked immunosorbent assay (ELISA) with the human PRL ELISA Kit (EC Bio Labs, Delhi, India).

Results

In healthy individuals, salivary and serum PRL levels were 4.26 ± 1.37 ng/ml and 13.08 ± 2.47 ng/ml, respectively. In patients with periodontitis, levels significantly declined post-therapy, from 8.96 ± 1.46 to 6.30 ± 1.28 ng/mL (saliva) and 13.09 ± 2.56 to 8.10 ± 1.34 ng/mL (serum). PRL levels showed a strong baseline correlation with clinical parameters, which persisted as a weak yet significant correlation post-treatment in the periodontitis group.

Conclusions

The findings of this study suggest that PRL can be a valuable biomarker for monitoring disease activity and treatment. Furthermore, the significant correlation between salivary and serum PRL levels suggests that PRL may be a cost-effective, minimally invasive marker linking oral and systemic health.

## Introduction

Periodontal disease is a complex infectious disease resulting from the interplay between bacterial infection and the host's response to bacterial challenge [1]. Bacterial factors can trigger a local inflammatory response and activate the innate immune system [2]. The inflammatory process involves a complex network of cytokines and chemokines [3]. Changes in the levels of circulating hormones have been reported to enhance gingival inflammation [4].

Prolactin (PRL), a 23-kDa peptide hormone, functions as a cytokine and is associated with the pathogenesis of various chronic inflammatory diseases [5]. PRL was originally named after its first discovered function, that is, the stimulation of milk production. However, this hormone has more than 300 functions, including actions on reproduction, osmoregulation, behavior, immune regulation, growth, and metabolism [6].

PRL secretion is upregulated by cytokines such as IL-1, IL-2, and IL-6, which are stimulated during inflammation, and inhibited by endothelin-3 and interferon-γ [7]. Although PRL is mainly synthesized in the anterior pituitary, the central nervous system, the immune system, the uterus, and the mammary glands are also capable of producing PRL [8]. Hyperprolactinemia has been detected in patients with autoimmune diseases like rheumatoid arthritis (RA), systemic lupus erythematosus (SLE), Sjögren’s syndrome, multiple sclerosis, autoimmune thyroid disease, and others [9]. Although the mechanisms underlying this interaction are not yet fully understood, it has been documented that PRL can influence the communication and regulation of immune cells [7].

Few studies have reported elevated levels of PRL in the gingival crevicular fluid (GCF) of patients with periodontitis compared to healthy controls, which have been found to decrease with periodontal therapy [10,11]. However, no studies have assessed the levels of PRL in human saliva in the context of periodontitis. Recent research is focusing on the development of saliva as a diagnostic fluid. Evaluating the levels of salivary PRL in periodontitis will serve two purposes: firstly, the role of salivary PRL as a potential biomarker in periodontitis can be assessed; secondly, the potential correlation between salivary and serum PRL levels may help establish a link between periodontal and systemic involvement, positioning PRL as an inflammatory mediator. This could pave the way for utilizing saliva as a non-invasive diagnostic medium for various systemic inflammatory conditions in the future. Therefore, the present study aims to estimate and correlate the salivary and serum PRL levels in patients diagnosed with periodontitis before and after non-surgical periodontal therapy, compared to healthy controls.

The primary objective of this study was to estimate and compare salivary and serum PRL levels in patients with generalized Stage II-IV periodontitis before and after non-surgical periodontal therapy, as well as in systemically healthy individuals with periodontal health. The secondary objectives were to evaluate the correlation between salivary and serum PRL levels, assess their association with clinical periodontal parameters, and explore the potential of salivary PRL as a non-invasive biomarker for periodontal inflammation.

## Materials and methods

Study design

This prospective comparative case-control study with longitudinal follow-up in the periodontitis group included 60 individuals aged between 25 and 50 years who reported to the Department of Periodontics, Vydehi Institute of Dental Sciences and Research Centre, Bengaluru. The study was approved by the Vydehi Institute of Dental Sciences Institutional Ethics Committee (approval number: VIDS-IEC/PG/APP/2023/61) and was conducted over a defined period from June 2023 to September 2024. Written informed consent was obtained from all participants in accordance with the Declaration of Helsinki (1964; amended 2024). Participant confidentiality and data protection were strictly maintained throughout the study. All participants had a minimum of 20 natural teeth. Group 1 comprised 30 systemically healthy individuals with no clinical signs of gingival inflammation, no clinical attachment loss, and probing depths ≤3 mm, while Group 2 consisted of 30 patients with generalized Stage II-IV periodontitis, as defined by the 2017 World Workshop on Periodontal and Peri-Implant Diseases and Conditions [12]. Patients were included if they exhibited interdental clinical attachment loss of ≥3 mm at the most affected site and a probing depth of >5 mm in at least 30% of the teeth. Exclusion criteria included systemic illness, oral lesions, pregnancy, oral contraceptive use, lactation, current or former tobacco use, prior periodontal therapy within the previous six months, use of antibiotics or anti-inflammatory drugs within the previous three months, or current pharmacological therapy that could affect periodontal status.

Clinical protocol

At baseline, unstimulated saliva and venous blood samples were collected, followed by the recording of clinical periodontal parameters. These included the Plaque Index (Silness and Löe, 1964) [13], the Gingival Index (Löe and Silness, 1963) [14], Bleeding on Probing [15], Probing Depth, and Clinical Attachment Level, measured at six sites per tooth using a UNC-15 periodontal probe (Hu-Friedy, Chicago, USA). Intra-examiner calibration was performed by a single experienced examiner, who repeated measurements in 10 patients at 48-hour intervals. Calibration was accepted when 90% of readings demonstrated concordance within ±1 mm. All participants underwent non-surgical periodontal therapy in the form of scaling and root planing (SRP), performed with ultrasonic and manual instruments under local anesthesia when required. Oral hygiene instructions, including the Modified Bass brushing technique and interdental cleaning aids, were provided and reinforced during follow-up visits. Patients in the periodontitis group were recalled after three months for repeat clinical evaluation and collection of saliva and serum samples.

Sample collection

Following the completion of patient recruitment and initial recording of clinical parameters, each participant was recalled the following day to minimize the potential risk of blood contamination. To reduce the influence of diurnal variation, unstimulated whole saliva samples (5 mL) were collected from each subject between 9:00 a.m. and 10:00 a.m. using the passive drool method. Participants were instructed to refrain from food consumption for at least one hour prior to sample collection. Seated in a relaxed, upright position on a dental chair, subjects were asked to allow saliva to pool in the floor of the mouth and then passively drool into a sterile collection container over a period of five minutes. The collected samples were centrifuged at 2500 rpm for five minutes. The resulting supernatant was carefully transferred into sterile plastic vials and stored at −80°C for subsequent analysis.

A 5 mL venous blood sample was collected from the antecubital fossa through venipuncture using a 20-gauge needle and a 5 mL syringe. The collected blood was allowed to clot by incubating it in an upright position at room temperature for 30 to 45 minutes, with a maximum incubation time of 60 minutes. The serum was then separated from the blood by centrifugation at 2500 rpm for five minutes. The resulting serum was immediately transferred into sterile plastic vials and stored at −80°C until further analysis. Saliva collection posed no risk to participants, while venipuncture for serum collection involved minimal risks, such as transient discomfort, bruising, or, rarely, infection. All procedures were performed by trained personnel under aseptic conditions to minimize these risks.

Treatment protocol

Following the initial clinical examination and sample collection, non-surgical periodontal therapy was provided to all subjects in the form of SRP, utilizing a combination of ultrasonic devices and manual instruments, including scalers and curettes (Hu-Friedy, Chicago, USA). This therapy was executed in a quadrant-wise manner. Local anesthesia was administered as needed to ensure patient comfort and facilitate meticulous debridement. Comprehensive oral hygiene instructions, including the use of interdental cleaning aids, were provided to all participants. Emphasis was placed on the modified Bass tooth brushing technique, which was reinforced at each follow-up visit. Participants in the periodontitis group were scheduled for a three-month follow-up appointment for clinical re-evaluation, during which post-treatment saliva and serum samples were collected.

Prolactin analysis procedure

An enzyme-linked immunosorbent assay (ELISA), the Human PRL ELISA Kit (EC Bio Labs, Delhi, India), was used to measure the pre- and post-treatment PRL levels. After the kit was equilibrated at room temperature, 100 μL of standard working buffer or 100 μL of sample was added into the appropriate wells and incubated at 37°C for 80 minutes. The liquid in the plate was discarded, and 200 μL of 1× wash buffer was added to each well. The plate was then washed three times. After patting it dry against clean absorbent paper, 100 μL of biotinylated antibody working solution (1×) was added to each well and incubated at 37°C for 50 minutes. Two hundred microliters of 1× wash buffer were added to each well after discarding the liquid in the plate, and the plate was washed three times. One hundred microliters of 1× Streptavidin-HRP working solution was added to each well and incubated at 37°C for 50 minutes after the well was dried with absorbent paper. The liquid in the plate was discarded again, and 200 μL of 1× wash buffer was added to each well, followed by five wash cycles. After patting dry, 90 μL of TMB substrate solution was added to each well and incubated at 37°C for 20 minutes in the dark. Fifty microliters of stop solution were added to each well, and the plate was shaken on a plate shaker for one minute. Optical density was recorded at 450 nm immediately, and the results were calculated. The detection range for PRL was 1.57 to 200 ng/mL.

Statistical analysis

The sample size estimation for this study was performed at a 5% alpha error (α = 0.05), with an effect size of 74% (based on the findings of previous literature [10]), and the study's power was set at 80%. This demonstrated that a minimum of 60 subjects were needed for the present study; thus, each group consisted of 30 participants.

The normality of the data distribution was verified using the Shapiro-Wilk test. As the variables followed a normal distribution, parametric tests (Student’s t-test, Pearson’s correlation) were applied, along with nonparametric tests where appropriate. Descriptive analysis of all explanatory and outcome parameters was performed using frequency and proportions for categorical variables and mean and standard deviation for continuous variables.

The Mann-Whitney test and chi-square test were used to compare intergroup mean age and gender distribution, respectively. The independent Student’s t-test was used to compare mean clinical parameters, salivary, and serum PRL levels between the two groups at baseline and three months. Intragroup comparison of the mean clinical parameters, salivary, and serum PRL levels between different time intervals in Group 2 was performed using the paired samples t-test. Pearson’s correlation test was used to assess the relationship between clinical parameters, salivary, and serum PRL levels at baseline and three months in each group. Stepwise multiple linear regression analysis was used to predict salivary and serum PRL levels at baseline and three months using clinical parameters in each group. The level of statistical significance was set at p < 0.05.

## Results

Participant flow

**Figure 1:**
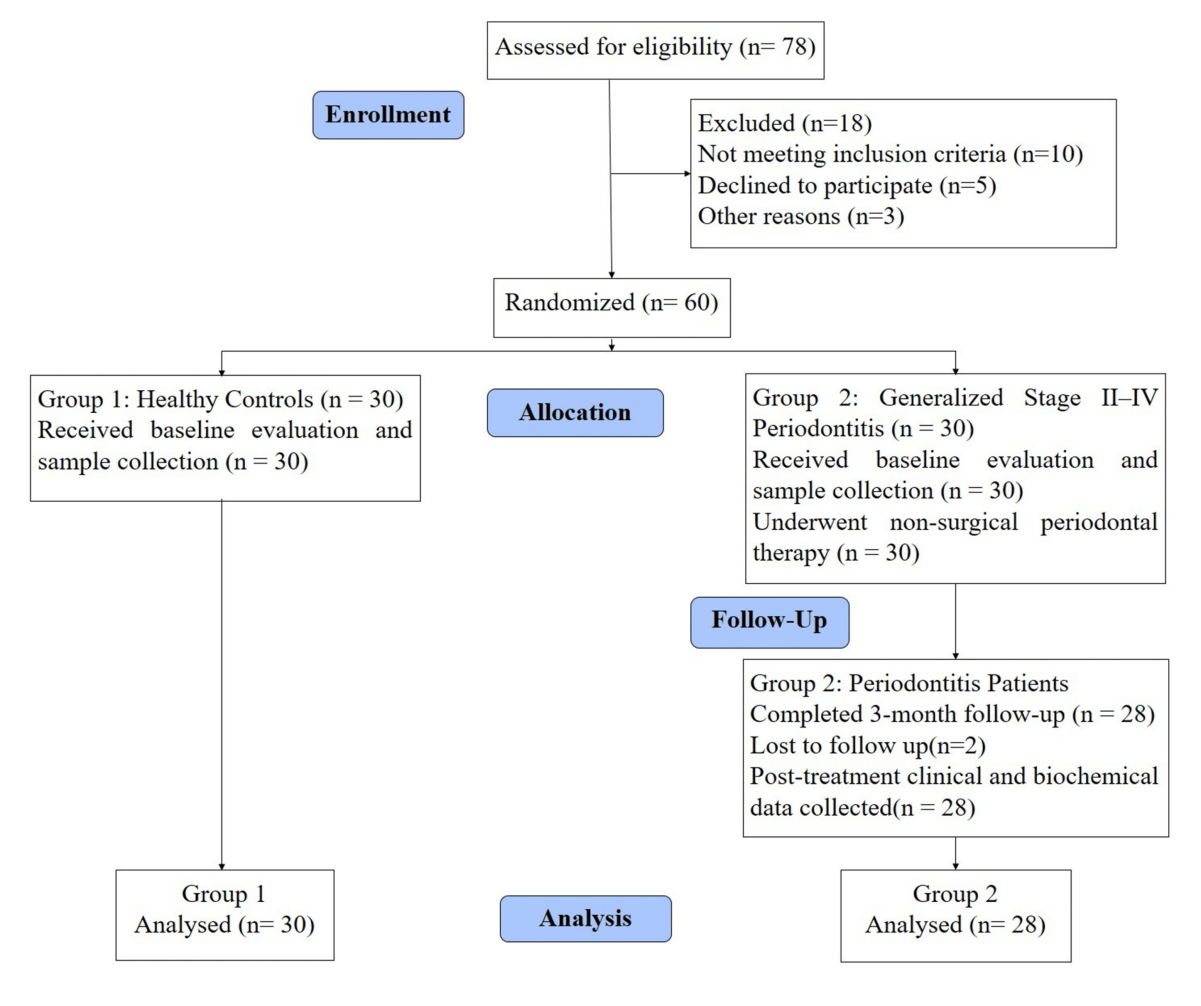
CONSORT flow diagram depicting the methodology including enrollment, allocation, follow-up, and analysis of subjects involved in the study

A total of 78 subjects were assessed for eligibility, of which 18 were excluded (10 did not meet the inclusion criteria, five declined participation, and three for other reasons). Sixty subjects were randomized into two groups: Group 1 (healthy controls, n = 30) and Group 2 (generalized Stage II-IV periodontitis, n = 30). All subjects underwent a baseline clinical evaluation, sample collection, and non-surgical periodontal therapy. At the three-month follow-up, 28 subjects in Group 2 completed the post-treatment evaluation, while two were lost to follow-up (patients relocated and could not report back). The final analysis included 30 subjects in Group 1 and 28 in Group 2 (Figure 1).

Demographic characteristics

The intergroup age and gender distribution (Table 1) demonstrated comparable mean values, with p > 0.05, indicating no statistically significant difference between the two groups.

**Table 1:** Age and gender distribution among the two groups The age distribution, measured with the Mann-Whitney test, and gender variation, measured with the chi-square test, show no significant difference between the two groups (p > 0.05). SD: standard deviation

Variable	Category	Group 1		Group 2		p-value
		Mean	SD	Mean	SD	
Age	Mean	34.97	4.21	36.43	4.46	0.13
	Range	29-45		28-46		
		n	%	n	%	
Gender	Males	18	60.0%	19	63.3%	0.79
	Females	12	40.0%	11	36.7%	

Clinical parameters

The intergroup comparison of clinical parameters at baseline (Table 2) revealed statistically significant differences (p < 0.001) across all measured variables (PI, GI, BOP, PD, and CAL).

**Table 2:** Intergroup comparison of mean values of clinical parameters and PRL levels at baseline period * statistically significant at p < 0.001. An independent Student's t-test was used to compare clinical parameters and PRL levels. GI: Gingival Index, PI: Plaque Index, BOP: Bleeding on Probing, PD: Probing Depth, PRL: prolactin, SD: standard deviation

Parameters	Group	N	Mean	SD	Mean diff	p-value
GI	Group 1	30	0.83	0.38	-1.60	<0.001*
	Group 2	30	2.42	0.33		
PI	Group 1	30	0.90	0.36	-1.57	<0.001*
	Group 2	30	2.47	0.31		
BOP	Group 1	30	8.93	0.96	-86.04	<0.001*
	Group 2	30	94.97	3.72		
PD	Group 1	30	1.69	0.43	-4.69	<0.001*
	Group 2	30	6.38	0.74		
Salivary PRL	Group 1	30	4.26	1.37	-4.70	<0.001*
(ng/mL)	Group 2	30	8.96	1.43		
Serum PRL	Group 1	30	6.85	1.86	-6.22	<0.001*
(ng/mL)	Group 2	30	13.08	2.47		

Non-surgical periodontal therapy in the periodontitis group resulted in statistically significant improvements across all measured variables (Table 3). Notably, PI, BOP, PD, and CAL demonstrated marked reductions with p < 0.001, while GI also showed significant improvement (p = 0.002). However, despite these improvements, the measured parameters in the periodontitis group remained elevated compared to those of the healthy group.

**Table 3:** Intragroup comparison of mean values of clinical parameters and PRL values between baseline and three months in Group 2 * statistically significant at p < 0.001. A paired samples t-test was used to compare the mean values of clinical parameters and PRL levels between the baseline and three-month time points in Group 2. GI: Gingival Index, PI: Plaque Index, BOP: Bleeding on Probing, PD: Probing Depth, CAL: Clinical Attachment Level, SD: standard deviation

Parameters	Time	N	Mean	SD	Mean diff	p-value
GI	Baseline	28	2.42	0.34	0.30	0.002*
	3 months	28	2.12	0.60		
PI	Baseline	28	2.47	0.32	1.00	<0.001*
	3 months	28	1.47	0.52		
BOP	Baseline	28	95.12	3.77	25.62	<0.001*
	3 months	28	69.50	10.23		
PD	Baseline	28	6.40	0.76	1.32	<0.001*
	3 months	28	5.07	0.79		
CAL	Baseline	28	6.05	0.93	1.07	<0.001*
	3 months	28	4.98	0.79		
Salivary PRL	Baseline	28	8.96	1.46	2.66	<0.001*
(ng/mL)	3 months	28	6.30	1.28		
Serum PRL	Baseline	28	13.09	2.56	4.99	<0.001*
(ng/mL)	3 months	28	8.10	1.34		

Biochemical analysis

**Figure 2:**
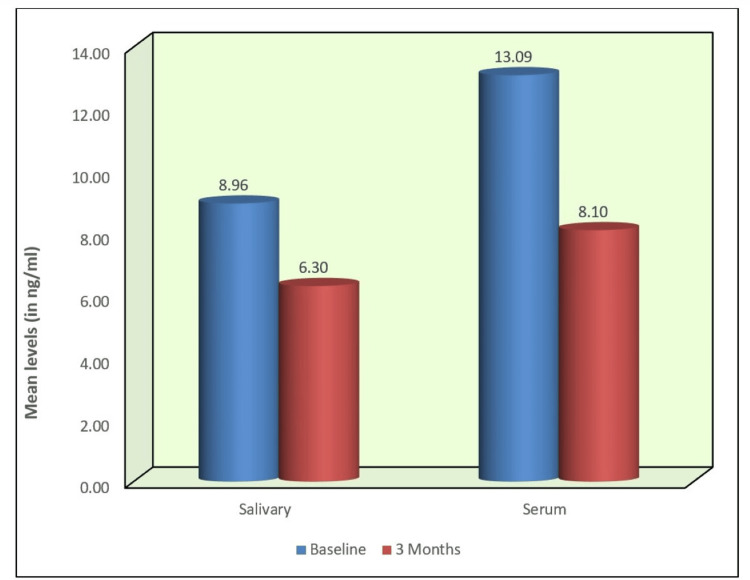
Salivary and serum PRL levels (ng/ml) between baseline and three months in Group 2 PRL: prolactin

Salivary and serum PRL levels were notably lower in Group 1. The periodontitis group exhibited considerably elevated mean salivary PRL and higher mean serum PRL values. The mean difference, with a p < 0.001 (Table 2), confirmed the statistically significant disparity in salivary and serum PRL concentrations between the groups. Non-surgical periodontal therapy in patients with periodontitis reduced PRL levels. A comparison of salivary and serum PRL levels between baseline and three months in this group demonstrated statistically significant (p < 0.001) reductions in both sample types (Figure 2, Table 3).

Correlation analysis

Pearson’s correlation analysis, evaluating the relationship between salivary and serum PRL levels and clinical parameters at baseline (Table 4), revealed that in Group 1, salivary PRL levels demonstrated a weak positive correlation with serum PRL, suggesting a mild but statistically significant association. However, correlations with GI, PI, BOP, PD, and CAL were weak and non-significant. Serum PRL levels, in contrast, exhibited a moderate negative correlation with GI and a weak negative correlation with PI, while correlations with other parameters, including BOP, PD, and CAL, were weak and non-significant.

**Table 4:** Pearson's correlation to assess the relationship between SaP and SeP levels and clinical parameters at baseline in each group and at three months in Group 2 * statistically significant at p < 0.001 SaP: salivary prolactin, SeP: serum prolactin, GI: Gingival Index, PI: Plaque Index, BOP: Bleeding on Probing, PD: Probing Depth, CAL: Clinical Attachment Level

Groups	Variable	Value	SeP	GI	PI	BOP	PD	CAL
Group 1 (baseline)	SaP	r	0.39	-0.13	-0.05	0.20	-0.03	-
		p-value	0.03*	0.51	0.81	0.28	0.89	-
	SeP	r	1	-0.52	-0.39	0.14	-0.27	-
		p-value	-	0.003*	0.04*	0.47	0.15	-
Group 2 (baseline)	SaP	r	0.68	0.64	0.64	0.57	0.65	0.66
		p-value	<0.001*	<0.001*	<0.001*	0.001*	<0.001*	<0.001*
	SeP	r	1	0.62	0.64	0.57	0.67	0.68
		p-value	-	<0.001*	<0.001*	0.001*	<0.001*	<0.001*
Group 2 (3 months)	SaP	r	0.71	0.67	0.63	0.67	0.60	0.68
		p-value	<0.001*	<0.001*	<0.001*	<0.001*	0.001 *	<0.001*
	SeP	r	1	0.71	0.67	0.70	0.70	0.73
		p-value	-	<0.001*	<0.001*	<0.001*	<0.001*	<0.001*

In Group 2, the baseline salivary and serum PRL levels demonstrated strong positive correlations across all measured clinical parameters (GI, PI, BOP, PD, and CAL), which were statistically significant, reinforcing the association between PRL concentrations and clinical deterioration.

Stepwise multiple linear regression analysis predicting salivary and serum PRL levels using clinical parameters at baseline in Group 2 (Table 5) highlighted that for every 1 mm increase in CAL, salivary and serum PRL levels increased by 1.044 ng/mL and 1.859 ng/mL, respectively, identifying CAL as the primary predictor for PRL levels.

**Table 5:** Stepwise multiple linear regression analysis to predict salivary and serum PRL levels using clinical parameters in each group at baseline and three months * statistically significant at p < 0.001 PRL: prolactin, DV: dependent variable, IV: independent variable, β: unstandardized beta, SE: standard error, t: t statistic, R2: correlation coefficient

Groups	DV	IV	Unstd. coefficients		t	p-value	R^2^
			β	SE			
Group 2 (baseline)	Salivary PRL	Constant	2.672	1.365	1.957	0.06	0.42
		CAL	1.044	0.224	4.659	<0.001*	
	Serum PRL	Constant	1.881	2.306	0.816	0.42	0.44
		CAL	1.859	0.379	4.909	<0.001*	
Group 2 (3 months)	Salivary PRL	Constant	0.806	1.178	0.684	0.5	0.44
		CAL	1.103	0.234	4.72	<0.001*	
	Serum PRL	Constant	1.902	1.161	1.638	0.11	0.51
		CAL	1.244	0.23	5.402	<0.001*	

Regression analysis at three months post-treatment in Group 2 (Table 5) also showed a statistically significant association between CAL and salivary PRL levels. Each 1 mm reduction in CAL corresponded to a 1.103 ng/mL decrease in salivary PRL levels, signifying a downward shift in PRL levels post-treatment. In serum, CAL accounted for 51% of the variance in PRL levels, with each 1 mm reduction in CAL associated with a 1.244 ng/mL decrease in serum PRL, reinforcing the inverse correlation between PRL concentration and periodontal improvement.

In the present study, mean serum PRL in the periodontitis group at baseline (13.1 ng/mL) was elevated compared with healthy individuals (6.8 ng/mL), approaching the upper limit of the normal reference range (5-20 ng/mL in females, 4-15 ng/mL in males). Following therapy, serum PRL levels declined by ~38% to 8.1 ng/mL, which is well within normal limits. Similarly, salivary PRL decreased from 8.9 ng/mL at baseline to 6.3 ng/mL post-treatment, which is lower than the reported salivary range of healthy controls (4.2 ng/mL) and aligns more closely with the reported salivary range of ~2-5 ng/mL.

## Discussion

Periodontitis pathogenesis stems from a complex host-microbe interplay, wherein bacterial components, such as lipopolysaccharides and peptidoglycans, activate toll-like receptors, triggering an inflammatory cascade [16]. Hormones modulate cellular functions pivotal to inflammation, including vascular responsiveness, directed cell migration, phagocytosis, and microbicidal activity [17]. PRL is a polypeptide hormone with pleiotropic functions, including immune reactivity, linking it to the pathogenesis of various inflammatory disorders [18,19]. The binding of PRL to its receptor activates downstream signaling pathways that regulate the proliferation, differentiation, secretion, and survival of immune cells [7].

Over the past two decades, accumulating evidence has linked PRL to immune regulation, underscoring significant crosstalk between the (neuro)endocrine and immune systems [18]. Elevated PRL concentrations have been observed during the active phases of various inflammatory and autoimmune diseases, such as Behçet’s disease and SLE, with positive correlations between PRL levels and inflammatory biomarkers noted exclusively in active disease states, but not in remission or in healthy individuals [19]. Thus, the present study was undertaken based on the hypothesis that PRL levels elevate in inflammatory conditions such as periodontitis, potentially serving as a valuable positive biomarker in affected individuals.

This study assessed salivary and serum PRL levels, along with clinical parameters, in healthy individuals and patients with Stage II-IV periodontitis, also evaluating post-treatment changes three months after SRP. The two groups demonstrated demographic homogeneity, with no statistically significant disparities in age or sex distribution. As PRL is primarily known for its role in lactotropin-releasing hormone, which is primarily associated with milk production in lactating females, gender variation plays a significant role in its distribution. Lundberg et al. [20] observed a female predilection for PRL, noting elevated serum levels in women with expansively growing pituitary adenomas. However, contrasting findings from other studies [10,21] reported no significant gender-based variation in this hormone. The present study also found no variation in PRL levels based on gender.

In the present study, baseline salivary and serum PRL levels were significantly elevated in patients with periodontitis compared to those with gingival health, corroborating the findings of El Wakeel et al. [10], who reported enhanced expression of GCF PRL concentrations in patients with periodontitis. Their subsequent study [11] further substantiated PRL’s role as a potential inflammatory nexus between periodontitis and RA, with elevated GCF and serum PRL levels in chronic periodontitis patients, both with and without RA, which declined post-NSPT yet remained higher than the current study’s findings. Conversely, Ozdemir et al. [22] reported no significant differences in PRL levels between patients with periodontitis and healthy controls, although they observed elevated PRL levels in patients with acromegaly. This difference may be attributed to systemic hormonal imbalance in acromegaly, the pleiotropic and multidirectional role of PRL across biological systems, and methodological discrepancies. Additionally, Jacobi et al. [23] demonstrated elevated serum PRL levels in SLE and other autoimmune conditions, correlating with disease activity, thereby reinforcing the involvement of PRL in immune-inflammatory dynamics. The present findings align with this paradigm, demonstrating increased levels of salivary and serum PRL in patients with periodontitis compared to healthy subjects.

Clinical parameters revealed significantly greater gingival inflammation, plaque accumulation, bleeding tendency, pocket depth, and clinical attachment loss in periodontitis patients compared to the healthy group, with marked improvement across all variables three months post-treatment. Substantial evidence in the literature supports NSPT in the form of SRP as the initial therapy before surgical interventions in severe periodontitis [23-25].

Alongside improvements in clinical parameters, the periodontitis group exhibited a notable reduction in salivary and serum PRL levels three months post-treatment. However, levels remained higher than those in the healthy group. Similarly, El Wakeel et al. [10] reported decreased GCF PRL levels following initial periodontal therapy, albeit with slightly higher post-treatment values compared to the present findings, likely due to differences in study design. Given the limited literature on salivary PRL in periodontitis, comparisons were restricted to studies utilizing GCF samples.

Clinical, animal, and in vitro studies collectively indicate that PRL possesses immunostimulatory properties, activating T cells, B cells, natural killer cells, macrophages, neutrophils, CD34+ hematopoietic progenitors, and dendritic cells [26]. Pro-inflammatory cytokines, such as IL-1 and TNF-α, key mediators in periodontal tissue destruction, are known to stimulate PRL secretion. Additionally, PRL directly impacts osteoblasts via PRLR expression, leading to reduced proliferation, increased apoptosis, and diminished calcium content, thereby impairing bone formation [27]. Its association with osteoporosis in both sexes is well documented [28]. Elera-Fitzcarrald et al. [29] reported a correlation between elevated serum PRL and reduced bone mineral content in SLE patients. Given that periodontitis involves inflammatory bone loss through impaired osteoblastic activity, PRL may play a contributory role in disease progression, suggesting its potential as a mechanistic link between periodontal and systemic inflammatory disorders.

Pearson’s correlation analysis revealed stronger associations between PRL levels and clinical parameters in patients with periodontitis compared to healthy individuals. The persistence of this correlation at three months post-treatment in periodontitis patients indicates a clear link between elevated PRL levels and periodontal disease severity. As CAL is a key indicator of periodontitis severity, its strong association with PRL underscores the hormone’s potential involvement in PRL-associated periodontal changes. At three months, the observed post-treatment decline in both CAL and PRL levels highlighted a significant inverse correlation between PRL concentration and periodontal improvement.

The consistent elevation of PRL levels in both serum and saliva during active periodontal inflammation, alongside their significant reduction following NSPT, underscores PRL’s potential as a dynamic and objective biomarker for disease activity. Its measurable fluctuations in tandem with clinical parameters, particularly CAL, suggest that PRL not only reflects disease severity but may also serve as a reliable indicator of therapeutic response. The use of saliva as a diagnostic medium enhances its clinical applicability, offering a non-invasive, accessible, and patient-friendly tool for real-time monitoring of periodontal status. Furthermore, given PRL’s established roles in immune modulation and bone metabolism, as well as its elevated levels in various systemic autoimmune and inflammatory disorders, its expression in periodontitis may also signal broader systemic immune dysregulation. This supports the growing view of periodontitis as a manifestation of systemic inflammatory burden and positions PRL as a promising biomarker linking oral and systemic health.

To the best of our knowledge, this is the first study to estimate salivary PRL levels and correlate them with serum PRL concentrations in the context of periodontitis, thereby introducing a novel, less invasive diagnostic approach. By excluding individuals with systemic conditions known to influence PRL levels, the study effectively minimized confounding variables. It allowed a more precise evaluation of periodontitis as an independent risk factor potentially linked to systemic inflammatory burden. The strong correlation between salivary and serum PRL levels underscores the utility of saliva as a surrogate diagnostic fluid, offering a real-time, chairside assessment of periodontal inflammation. However, the limitations of this study include potential confounding factors that may influence PRL levels, such as menstrual cycle phase, stress, and sleep patterns, which were not specifically controlled for. In addition, the relatively small sample size and short follow-up duration highlight the need for larger, multi-center studies across diverse populations to validate and generalize these findings.

## Conclusions

This study suggests that PRL may serve as a potential biomarker in both saliva and serum, reflecting the state of periodontal health. Elevated PRL levels in periodontitis patients correlated positively with clinical parameters, highlighting its association with disease severity. Non-surgical periodontal therapy significantly reduced PRL levels, underscoring its role in both disease progression and resolution. While the interventional design strengthens the reliability of these findings, conclusions regarding PRL’s biomarker value should be drawn cautiously. Further large-scale, longitudinal studies are necessary to validate PRL as a consistent and clinically applicable marker of periodontal and systemic inflammatory status.
